# Innate immune activation and white matter injury in a rat model of neonatal intraventricular hemorrhage are dependent on developmental stage

**DOI:** 10.21203/rs.3.rs-2512127/v1

**Published:** 2023-01-27

**Authors:** Miriam Zamorano, Scott D. Olson, Candice Haase, Charles S. Cox, Brandon A. Miller

**Affiliations:** University of Texas Health Science Center at Houston; University of Texas Health Science Center at Houston; University of Texas Health Science Center at Houston; University of Texas Health Science Center at Houston; University of Texas Health Science Center at Houston

**Keywords:** Intraventricular hemorrhage, hydrocephalus, innate immunity, white matter, oligodendrocyte

## Abstract

**Background:**

Inflammation and white matter injury are consequences of neonatal intraventricular hemorrhage (IVH). Both white matter and the neuroimmune system are developing during which IVH and its consequences occur. IVH has been studied in many different animal models; however, the effects of IVH occurring at different developmental time points in the same model has not been examined. Examining how the timing of IVH affects the ultimate outcome of IVH may provide important insights into IVH pathophysiology.

**Methods:**

We used intraventricular injection of lysed whole blood to model neonatal IVH in postnatal day (P)2 and P5 rats. Flow cytometry was used to detect innate immune activation. MRI was used to screen animals for the development of increased ventricular size. Immunohistochemistry for myelin basic protein was used to assess white matter pathology.

**Results:**

The acute response of the innate immune system at these time points differed, with P5 animals exhibiting significant increases in several measures of classically pro-inflammatory innate immune activation that P2 animals did not. Animals with IVH induced at P5 also developed ventricular enlargement visible on MRI whereas animals with IVH induced at P2 did not. On histological analysis, there were no significant effects of IVH in P2 animals, but IVH in P5 animals induced a reduction in several measures of white matter integrity.

**Conclusions:**

IVH induces a strong innate inflammatory response in P5 animals that correlates with changes in ventricular size and white matter. P2 animals did not exhibit any significant changes in innate immune activation or white matter structure after IVH. This suggests that the white matter pathology from IVH is due in part to innate immune activation; and that the developmental stage of the innate immune system is a key determinant of IVH pathology.

## Background

Neonatal intraventricular hemorrhage (IVH) is a common complication of preterm birth. IVH activates innate immune cells and injures developing white matter [1, 2]. IVH occurs during the early postnatal period, during which both the central nervous system and immune system are developing. Previously, we used intraventricular injection of hemoglobin (Hgb) in post-natal day (P)5 rat pups to model inflammation and white matter injury occurring after neonatal IVH [3]. This is consistent with other studies that used this developmental stage [4–6], though some groups have modeled IVH using younger animals [7]. These differences are important because the developmental stage of oligodendrocyte progenitor cells (OPCs) determines how white matter responds to injury [8, 9]. An ideal model of neonatal IVH should be performed at the stage of brain development that best represents that of a preterm infant. However, a range of post-natal rat brain ages may resemble the white matter of a preterm infant [10, 11]. Although the stepwise nature of white matter development has been appreciated for some time [12, 13], neuroimmune development is less well understood. New data have shown that microglial phenotype changes across the lifespan of an organism [14] and that different developmental stages of microglia have specific functions such as promoting myelination [15]. There is a dearth of understanding as to how neuroimmune development affects the pathology that occurs during periods of rapid brain and immune development.

Our prior IVH model, utilizing Hgb injection, allowed us to study the inflammatory pathology of IVH specifically mediated by Hgb [16]. Hgb can be easily delivered *in vitro* and does not confound biochemical assays for proteins produced by immune cells that are present in whole blood. However, there are other components of whole blood; such as plasmin [17, 18], complement [19], thrombin[18], fibrinogen[20], platelet releaseate [21], and even albumin [22], that can affect white matter development [23], just as Hgb does [24]. Others have injected whole blood into [25] or near [26, 27] the ventricular system in rodents and whole blood components have been tested in developing CNS cells *in vitro* with deleterious effects [18, 24]. Therefore, in this study we utilized lysed whole blood injection to better mimic the mix of substances that affect inflammation and white matter injury in human IVH.

This study was designed to examine inflammation and white matter injury across a narrow and developmentally important age range in response to IVH. We hypothesized that IVH occurring at an earlier developmental stage would lead to worse white matter pathology; however, this was not the case here. We found that the innate immune response of older animals was more robust, and that IVH occurring later in development resulted in more severe histological changes. White matter changes after IVH induced in older animals were evident throughout the brain, not just near the ventricles, suggesting that the effects of IVH are widespread. These data are the first to show that the outcome of IVH is critically dependent on the developmental stage of the brain and suggest that white matter injury after IVH is dependent on a strong innate immune response that is not present at earlier developmental stages.

## Materials And Methods

### Animals and surgical procedures

Timed pregnant Sprague Dawley rats were purchased from Charles River Laboratories (Wilmington, MA, USA) and housed in a climate-controlled room on a 14/10-hour light/dark cycle with food and water provided ad libitum. Post-natal day (P)2 and P5 rat pups were anesthetized via hypothermia with their skin protected by aluminum foil. Their heads were secured with an acrylic holder to prevent movement. The scalp was cleaned with betadine and the coordinates were visualized by transillumination of cranial sutures. Injection coordinates for P2 animals were 3.8mm from lambda, 1mm lateral and 2mm deep. For P5 animals, injection coordinates were 4.6mm from lambda, 1.2mm lateral and 3.3mm deep [28]. A 30G needle was gently placed directly through the scalp and skull and 30μl of lysed blood from a single female donor rat in each cohort was injected into the right ventricle at a flow rate of 0.5μl/sec. This injection volume was the same in P2 and P5 animals. 30μl of sterile isotonic saline injection was used as a control. The needle was left in place for 2 minutes after injection to prevent retrograde flow from the syringe tract. After injections, animals were returned to their home cage and dam for recovery.

### Cell isolation and flow cytometry

Flow cytometry was utilized to assess changes in the cellular immune response in treated animals similar to our previous work [29–31]. 24 hours after intraventricular lysed blood or saline injection as described above, animals were sacrificed by rapid decapitation and brains were removed. Meningeal tissue was removed and cortices were extracted and placed in DMEM (Invitrogen). Tissue was minced and centrifuged at 300g for 5 minutes and cells were resuspended in TryplE on an orbital shaker for 15min at 37°C. Cells were resuspend in DMEM/DNase solution and triturated with a glass pipette and resuspended in running buffer MACS buffer (Miltenyibiotec).

A single cell suspension was generated with a 40μm cell strainer and the suspension was centrifuged at 850g for 15 min at 4°C in 23% percoll. Cells were then washed and stained with Ghost v450 dye (1:1000, Tonbo, 13–0863-T100) for 30 min at 4°C. Cells were washed with FACS buffer (1% BSA in PBS) and supernatant aspirated after a 300g spin for 5min at 4°C. Cells were blocked using FcR blocking reagent CD32 (1:100, BD Pharmingen, 550271) for 5min at room temperture following staining with fluorescent antibody panels (CD45-PerCP-Cy5.5 (Biolegend), CD11b-APC-Cy7 (ThermoFisher), CD68-BV421 (BD Biosciences)) for 1 hr at 4°C and then washed twice. Data was acquired on a Gallios flow cytometer (Beckman Coulter) and analyzed using Kaluza vr. 2.1 and FlowJo vr. 10.8.1.

Events suitable for analysis were gated based on morphometric properties and for single cells based on forward scatter properties and then cells were assayed for mean fluorescence intensity for staining of CD45, CD11b, and CD68. Values were normalized to the saline-treated controls to measure relative mean fluorescence intensity (rMFI) specific to each experimental cohort. Expression of CD45 was used to establish CD45-negative, CD45-mid, and CD45-high gates to further evaluate expression of CD11b and CD68.

### MRI

T2 MRI images were obtained 5–6 days after IVH induction. Animals were anaesthetized with 3–4% isoflurane in a gas mixture of 30% oxygen and 70% medical air (flow rate 1.0 L/min) during the induction phase and then with 1–2% isoflurane during imaging. Body temperature and respiration were monitored with a physiologic monitoring system (Small Animal Instruments, Stony Brook, NY, USA). MRI was conducted on a 7T Bruker BioSpec system (Bruker Biospin, Billerica, MA, USA) equipped with a B-GA12 gradient at our imaging core facility. Images were acquired using a volume coil for excitation and a mouse brain surface coil for signal reception. After animal setup, T_2_-weighted anatomical imaging was acquired with RARE sequence using the following imaging parameters: TR: 6000ms, TE: 60ms, RARE factor: 8, matrix size: 128 × 128, FOV: 19.2 mm × 19.2mm, with a final resolution of 150 × 150mm^2^ and 0.5 mm slice thickness. After recovery from anesthesia pups were returned to their home cage and dam.

### Immunostaining and immunofluorescence

Animals were euthanized with isoflurane overdose. Brains were removed and fixed in 4% paraformaldehyde solution in 0.1 M sodium phosphate buffer PBS (pH 7.3) overnight then immersed in 20% sucrose for 1 day and then 30% sucrose for 3 days. Brains were then frozen in optimal cutting temperature compound (O.C.T., Sakura Finetek, Torrance, CA). 20μm sections were in the coronal plane. Slides were incubated with Anti-Myelin Basic Protein antibody polyclonal antibody (ab40390, Abcam) at 1:200 followed by secondary antibody incubation in Alexa-594 goat anti-rabbit at 1:500 (Invitrogen, Grand Island, NY).

### Image analysis

Slides were scanned into image files using a Nikon TiU Inverted motorized microscope equipped with 4–40x long working distance objectives (PlanFluor), Phase imaging, Nikon LS-2 color camera, and Zyla camera with DAPI, FITC, Texas Red, mCherry, and CFP filter sets. Measurements were made in ImageJ (NIH). Density was measured after converting images to grayscale and using the mean gray value measurement function. Density measurements were normalized to control values and reported as percent of control.

### Statistical analysis

Student’s t-test was performed to compare differences between groups using GraphPad v9.3.1. Because experiments in P2 and P5 animals were performed as separate experimental cohorts, statistical tests were run within the P2 and P5 cohorts but not used to directly compare data obtained from P2 and P5 animals. All data are reported as mean of the group ± SEM. Images used in figures are from subjects closest to the statistical mean for their group.

## Results

### Flow cytometry

**Figure 1:**
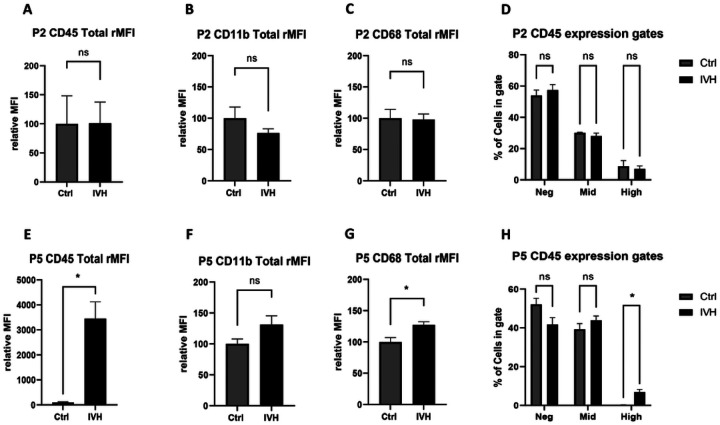
Innate immune activation after IVH in P2 and P5 animals. **A-C** CD45, CD11b and CD68 MFI did not differ in P2 animals after IVH. **D** CD45 expression gates did not differ in P2 animals after IVH. **E** CD45 MFI increased in P5 animals after IVH. **F** CD11b did not differ in P5 animals after IVH. **G** CD68 MFI increased in P5 animals after IVH. **H** CD45 high cells increased in P5 animals after IVH. *=p<0.05, n=4–5 per group.

Flow cytometry performed 24 hours after IVH was used to evaluate the innate immune response within the brain. When IVH was induced at P2, there was no statistical difference in the expression of CD45, CD11b, or CD68 fluorescence intensity between control or IVH groups (Fig. 1A, B, and C, p>0.05, n=4–5 per group for all). Similarly, there was no change in the number of CD45-mid cells, which are conventionally associated with microglia, or CD45-high cells, which are often associated with peripheral myeloid cells, between control and IVH groups (p>0.05 for all, Fig. 1D). We observed no significant differences in mean fluorescence intensity of CD11b or CD68 expression between the different CD45 expression-gated populations (Supplemental Figure 1, expression gates depicted on CD45 histograms, center column, in Supplemental Figure 2).

In contrast, IVH in P5 rats induced several significant differences in innate immune response. We found that IVH induced a marked increase in total CD45 expression (3454 ± 672 for IVH versus 100 for control, p<0.005, n=4–5 per group, Fig. 1E) a non-significant increase in CD11b expression (131 ± for IVH vs 100 for control, p=0.09, n=4–5 per group, Fig 1F) and a significant increase in overall CD68 expression (127 ± 14 for IVH vs 100 for control, p<0.05, n=4–5 per group, Fig. 1G). When separated into CD45-negative, CD45-mid, and CD-45 high-expressing subpopulations (gating depicted in Supplemental Figure 3, center column), we observed a significant increase in CD45-high cells (p<0.005, n=4–5 per group, Fig. 1H) but not CD45 mid cells (p=0.25, n=4–5 per group, Fig. 1H). Similarly, there was a significant increase in the expression of CD11b across each CD45-expressing subpopulations (p<0.05, Supplemental Figure 1).

**Figure 2:**
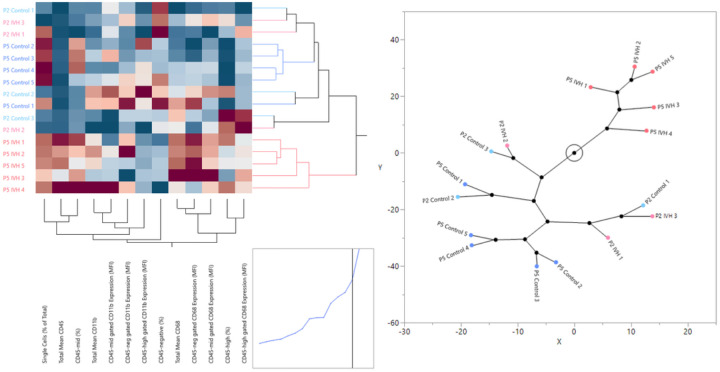
Constellation plot revealing clustering of experimental groups. P5 IVH animals cluster separately from P5 control and P2 control and IVH groups.

The combined measurements acquired from the P2 and P5 cohorts were normalized to their respective controls and analyzed for the relatedness of samples and measurements using two-way hierarchical clustering to generate a constellation plot to illustrate clustering of groups (Fig. 2). The clustering indicated that P2 IVH and control animals were interspersed and closely resembled P5 control animals, while P5 IVH animals exclusively clustered together indicating that the innate immune response of P5 IVH differed from that of the other groups.

### *in vivo* MRI analysis

**Figure 3:**
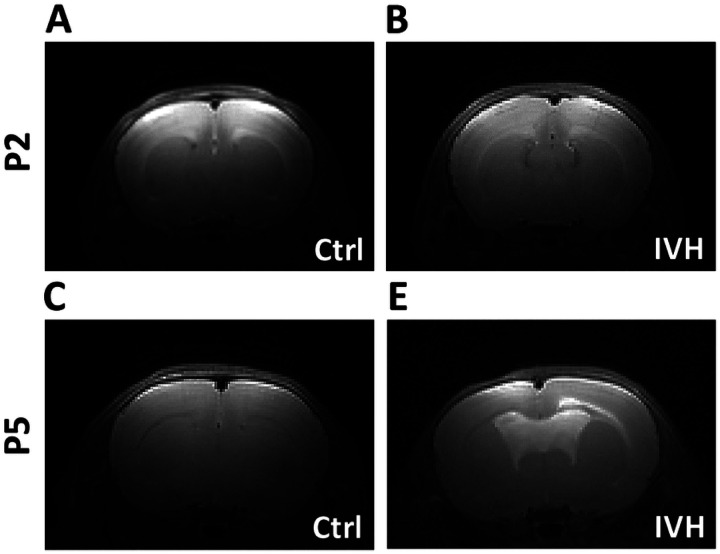
Brain MRI after IVH demonstrating increased ventricular size after IVH in P5 but not P2 animals. **A** Representative P2 control brain **B** Representative P2 IVH brain **C** Representative P5 control brain **D** Representative P5 IVH brain. *=p<0.05, n=7–9 per group.

MRI was performed in anesthetized animals 5 or 6 days after IVH or control injection for both P2 and P5 cohorts. MRI images were evaluated by a blinded observer for the presence of ventricular enlargement. No P2 control animals (n=7) were scored as having ventricular enlargement. One out of eight (13%) P2 IVH animals was scored as having ventricular enlargement. No P5 control animals were scored as having ventricular enlargement (n=7), and six out of seven (86%) P5 IVH animals were scored as having ventricular enlargement. The difference in the development of ventricular enlargement after IVH in P5 animals was statistically significant (p=0.0012, chi-square test). Representative images are shown in Figure 3. Ventricles in saline-injected animals were too small for precise measurement, therefore we quantified ventricle size via histology at the terminal time points.

### Ventricular size via histological measurement

**Figure 4:**
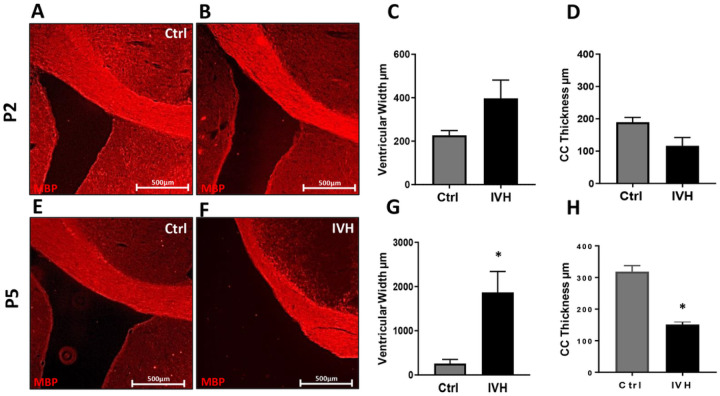
MBP histology showing ventricles and corpus callosum. **A-D** IVH in P2 animals did not induce significant changes in ventricular width or corpus callosum thickness. **E-H** IVH in P5 animals induced a significant increase in ventricular width and decrease in corpus callosum thickness. *=p<0.05, n=4–5 per group.

Ventricular width was measured in MBP-stained sections taken at the terminal time point for both P2 and P5 groups, P56 and P26, respectively (Fig. 4, A, B, E, F). Ventricular width was measured ipsilateral to the injection site in the first coronal section, where the third ventricle was visible. These effects were qualitatively similar on both sides of the brain. In P2 control animals, the average ventricular width was 227 ± 22μm and in P2 IVH animals the average ventricular width was 397 ± 84μm, which was not statistically significant (p=0.08, n=4–5 per group, Fig. 4C). In saline-injected P5 animals, the average ventricular width was 258 ± 93μm, and P5 IVH animal average ventricular width was 1869 ± 473μm, which was statistically significant (p=0.01, n=4–5 per group, Fig. 4E).

### Periventricular white matter analysis

We analyzed thickness of the corpus callosum ipsilateral to the side of injection in the same MBP-stained sections used for ventricular measurements. There was a statistically significant reduction in corpus callosum thickness in P5 IVH animals but not in P2 IVH animals. In P2 control animals, the average corpus callosum thickness was 189 ± 15μm, and in P2 IVH animals, average corpus callosum thickness was 116 ± 26μm (p=0.43, n=4–5 per group, Fig. 4D). In P5 control animals, average corpus callosum thickness was 318 ± 19μm and in P5 IVH animals the average corpus callosum thickness was 151 ± 9μm (p<0.005, n=4–5 per group, Fig. 4G).

**Figure 5:**
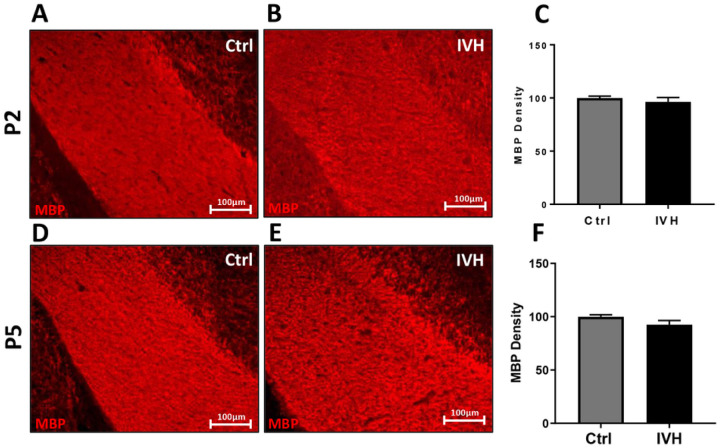
MBP density in the corpus callosum after IVH. **A-C** IVH in P2 animals did not induce a significant reduction in MBP density. **D-F** IVH in P5 animals did not induce a significant reduction in MBP density. n=4–5 per group.

We analyzed the density of white matter in the corpus callosum by selecting a section of white matter just lateral to midline on the ipsilateral side of injection and quantifying the average signal above a user-set threshold in this area (Fig. 5A, B, D, E). Unlike corpus callosum thickness, there was not a statistically significant reduction in MBP density in the corpus callosum in either P2 or P5 IVH animals when compared to controls. In P2 IVH animals, the average corpus callosum MBP density was 96±4% of control (p=0.4, n=4–5 per group, Fig. 5C). In P5 IVH animals and the average corpus callosum MBP density was 93±4% of control (p=0.15, n=4–5 per group, Fig. 5F).

### Peripheral white matter analysis

**Figure 6:**
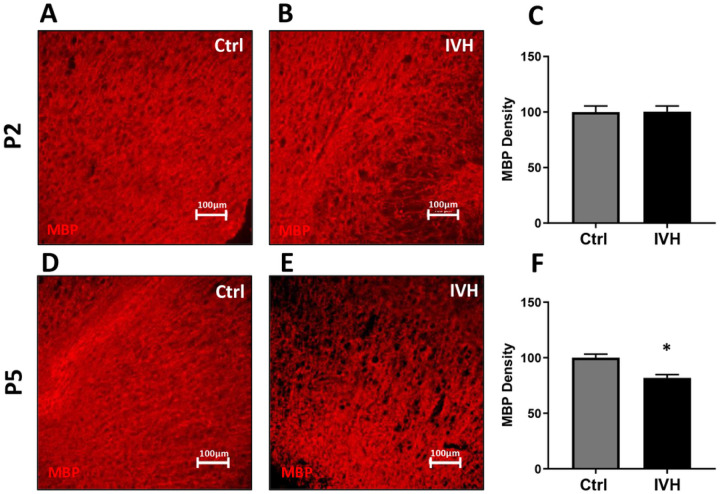
MBP density in the corona radiata after IVH. **A-C** IVH in P2 animals did not induce a significant reduction in MBP density. **D-F** IVH in P5 animals induced a significant reduction in MBP density. *=p<0.05, n=4–5 per group.

**Figure 7:**
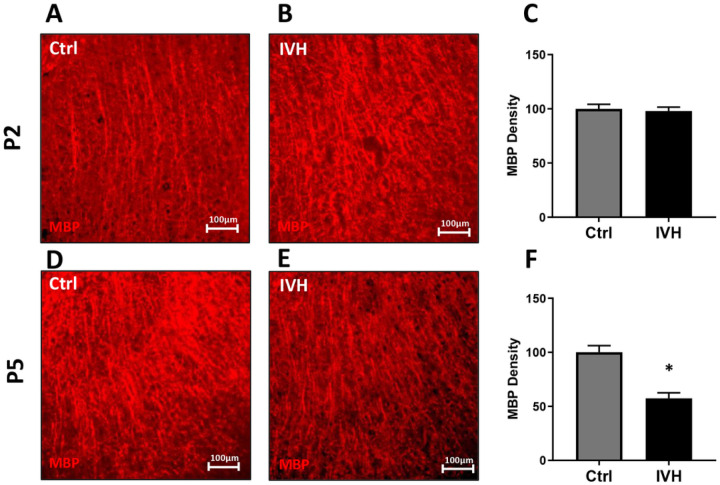
MBP density in the claustrum after IVH. **A-C** IVH in P2 animals did not induce a significant reduction in MBP density. **D-F** IVH in P5 animals induced a significant reduction in MBP density. *=p<0.05, n=4–5 per group.

To assess for more widespread effects of IVH on white matter structure, we analyzed MBP density in the corona radiata and claustrum in the same manner as in the corpus callosum (Fig. 6A, B, D, E) IVH at P5 but not P2 reduced MBP density in both the corona radiata and claustrum. In P2 IVH animals, the average corona radiata MBP density was 100±5% of control (p=0.96, n=4–5 per group Fig. 6C). In P5 IVH animals, average corona radiata MBP density was 82±3% of control (p<0.005, Fig. 6F). In P2 IVH animals, average claustrum MBP density was 98±4% of control (p=0.72, n=4–5 per group, Fig 7A, B, C). In P5 IVH animals, average claustrum MBP density was 57±5% of control (p<0.005, n=4–5 per group, Fig. 7D, E, F).

## Discussion

Here, we demonstrated that P5 rat pups mount a more pro-inflammatory innate immune response to IVH and sustain greater white matter injury than P2 pups. This is a particularly robust result, considering that we delivered the same volume of lysed blood into each age animal, resulting in a comparatively larger dose for P2 animals. Our flow cytometry data captures effects throughout the brain, and our previous work has shown that the acute inflammatory response to unilateral IVH is similar on both sides of the brain [3]. In this study, we examined only one time point, but our and other’ prior work showed that inflammation peaks early after IVH when both cytokine analysis and immunohistochemistry are used to quantify the innate immune response [3, 6]. This is similar to a large body of work from stroke and neurotrauma, where the acute inflammatory response peaks early after injury [32, 33]. The markedly different innate immune responses in P2 and P5 animals could be from either endogenous microglia, infiltrating macrophages, or both. However, these experiments were not designed to specifically test whether one type of innate immune cell was the largest contributor to this response. Delineating the differences between endogenous microglia and infiltrating macrophages in the context of development and injury is not straightforward. Antibodies that selectively label microglia may not be reliable in the early developmental period when these experiments were conducted [34] and may be altered by pathology [35, 36]. Furthermore, transient peripheral macrophage infiltration into the brain occurs under normal conditions during the first week of life [37]. Future work will attempt to better distinguish the relative contributions of microglia and peripheral macrophages to the different inflammatory responses detected in P2 and P5 animals. However, we postulate that this is the overall balance of pro-inflammatory cytokines and physiological growth factors that determines white matter fate after IVH rather than the particular cellular source of those factors.

Although white matter injury can be mediated by innate immune cells [8], developing oligodendrocytes are susceptible to direct injury by blood breakdown products [24]. The earlier the developmental stage of the oligodendrocyte, the more susceptible it is to injury from multiple insults [9, 38, 39]. Therefore, our initial hypothesis was that P2 animals would sustain worse white matter injury than P5 animals would. However, we observed more severe white matter pathology in the P5 animals. This suggests that the developmental stage of the innate immune system may be a more critical factor in determining the final white matter pathology after IVH than the developmental stage of white matter. The entire neonatal immune system differs from that of the adult, with an overall dearth of adaptive immune mechanisms compared to mature organisms [40]. Neonates who are born prematurely undergo the transition from an in utero immune system to a more mature immune system in an artificial environment, subject to physiological stressors that can lead to IVH. Clinical experience indicates that hydrocephalus and white matter injury after IVH evolve slowly over time [41]. Microscopically, events that lead to white matter injury are multifactorial and complex. Many molecules present in blood can be toxic to white matter, both directly by interacting with the cell membranes and receptors on OPCs, and indirectly by activating immune cells [1]. The source of these molecules is the intraventricular blood clot that is deposited at the margin of the ventricle at the ictus of IVH. Serial cranial ultrasound studies that are performed routinely in patients with IVH show that these clots gradually lyse and resolve. Therefore, there is likely to be a slow release of injurious factors over time in human IVH. While it is intuitive that the cumulative effects of blood products over time contribute to the ultimate pathology of IVH, our results suggest that the gradual maturation of the immune system over time may also contribute to IVH pathology. If this is the case, a better understanding of how the immune system operates at different ages could help direct surgical and pharmacologic therapy for IVH.

All of our measurements of white matter integrity showed that IVH at P5 induced white matter injury except for the corpus callosum MBP density. Interestingly, our prior work has shown that immunohistochemistry for MBP in the corpus callosum, may not be as sensitive as other measures of white matter integrity such as Western blot.[16] There may also be confounding factors that affect white matter analysis in the corpus callosum but not more peripheral white matter. It is possible that the corpus callosum was depleted of myelin, but due to compression from enlarged ventricles, a reduced amount of MBP was concentrated in a smaller cross-sectional area, making our density measurement less precise. Or the converse maybe true – that the corpus callosum was not significantly depleted of myelin (therefore no change in density), but was stretched as the ventricle enlarged (therefore reduction in corpus callosum thickness). Regardless of the one measure of white matter that was unchanged in the P5 IVH group, our results clearly demonstrate that IVH induced at P5 causes white matter injury that does not occur when IVH is induced at P2.

In addition to white matter injury, IVH also induces hydrocephalus. Most neurosurgeons consider the term “hydrocephalus” to signify increased intracranial pressure from pathological accumulation of CSF. Since we did not study CSF outflow, turnover, or intracranial pressure in this study, it is impossible for us to determine whether our rats had hydrocephalus or just increased ventricular size from white matter loss, as both brain volume loss and hydrocephalus can lead to large ventricles. Therefore, we used the terms “ventricular size” or “ventricular width” to describe the anatomic findings in our study. Future work studying CSF flow will be better able to determine to what extent ventricular changes are due to white matter loss versus obstruction of CSF outflow from the ventricular system. The ventricular enlargement observed here appears more pronounced on histological analysis, likely due to artifacts produced by tissue fixation and slide preparation. On MRI, P2 and P5 control animals, and P2 IVH animals have ventricles that are so small that they cannot be reliably measured. P5 animals that undergo IVH have lateral ventricles that would be considered moderately dilated if compared to human neonates with IVH.

Hydrocephalus and white matter pathology overlap, but are not identical, phenomena as others have eloquently stated [2]. Treating hydrocephalus in animal models can restore white matter integrity [42], suggesting that high intracranial pressure from untreated hydrocephalus may further white matter injury that is not directly caused by inflammatory or cytotoxic effects of blood. Untangling the relative contribution of brain tissue loss and altered CSF dynamics is difficult in experimental IVH models, and even more so clinically [43]. Our results suggest that; the developing neuroimmune milieu may even help to alter the relative contribution of one phenomenon over the course of the disease in individual patients. Improved therapy for IVH will be driven by a better understanding of the pathophysiology of the disease but will also be depend on the acceptance that many complex multisystem diseases are successfully treated via immunomodulatory therapy even before their pathophysiology is completely understood [44].

## Conclusions

Taken together, these results indicate that IVH induced in P5 rats produces a more robust innate immune response than IVH induced at P2. This could be due to different behavior of endogenous microglia or a more robust response of infiltrating macrophages at P5. IVH induced at P5 also leads to white matter and ventricular changes: therefore, it is likely that the difference in innate immune activation is linked to the long-term outcomes after IVH. Future studies on modulating innate immune activation could improve the outcomes after IVH.

## Data Availability

All data was collected at our center and is maintained per institutional guidelines
